# Influence of casein on the degradation process of polylactide-casein coatings for resorbable alloys

**DOI:** 10.1038/s41598-024-69956-6

**Published:** 2024-08-15

**Authors:** Katarzyna Cesarz-Andraczke, Marcin Staszuk, Tansel Tunçay, Anna Woźniak, Weronika Smok, Badegül Tunçay

**Affiliations:** 1 Department of Engineering Materials and Biomaterials, Faculty of Mechanical Engineering, Silesian University of Technology, Gliwice, Poland; 2 Manufacturing Engineering Department, Technology Faculty, Karabuk University, Karabuk, Turkey; 3 Mechanical Engineering Department, Engineering Faculty, Karabuk University, Karabuk, Turkey

**Keywords:** Biomedical materials, Structural materials

## Abstract

This study used the dip-coating method to develop a new biocompatible coating composed of polylactide (PLA) and casein for ZnMg1.2 wt% alloy implants. It evaluated its impact on the alloy's degradation in a simulated body fluid. After 168 h of immersion in Ringer's solution, surface morphology analysis showed that the PLA-casein coatings demonstrated uniform degradation, with the corrosion current density measured at 48 µA/cm^2^. Contact angle measurements indicated that the average contact angles for the PLA-casein-coated samples were below 80°, signifying a hydrophilic nature that promotes cell adhesion. Fourier-transform infrared spectroscopy (FTIR) revealed no presence of lactic acid on PLA-casein coatings after immersion, in contrast to pure PLA coatings. Pull-off adhesion tests showed tensile strength values of 7.6 MPa for pure PLA coatings and 5 MPa for PLA-casein coatings. Electrochemical tests further supported the favorable corrosion resistance of the PLA-casein coatings, highlighting their potential to reduce tissue inflammation and improve the biocompatibility of ZnMg1.2 wt% alloy implants.

## Introduction

Nowadays, the aging population and rising skeletal disorders prompt extensive research for advanced implants. As per the information outlined in the Global Burden of Disease Study 2021^1^, the worldwide prevalence of musculoskeletal disorder prevalence surged by 123.4% from 1990 to 2020, with projections anticipating a 115% increase by 2050. This rising trend drives demand for medical implants, highlighting the need for enhanced manufacturing and implant quality^2^. In the subject literature magnesium (Mg), iron (Fe), and zinc (Zn) based alloys are often tested for new resorbable implants, such as blood vessels stents or plates, screws, and nails for stabilizing fractured bones^3–7^. Mg-based alloy exhibit superior biocompatibility and appropriate mechanical properties. Magnezix alloy resorbable implants are already available on the market^8^. However, magnesium-based alloys degrade too rapidly (electronegative potential of − 2.37 V), and that proves challenging in the biodegradable implants field^9^. As Dong et al.^6^ indicated, the massive accumulation of hydrogen around Mg-based implants also impedes local tissue healing and reconstruction. In turn, for Fe and Zn-based materials, the percentage of hydrogen evolution vs. oxygen reduction is lower compared to that for Mg-based materials. Fe-based alloys, while biocompatible and strong, degrade too slowly (electronegative potential of − 0.44 V) and may cause cytotoxicity. As shown by Wegener et al.^7^ when the Fe ion concentration exceeds a critical value, it exhibits possible cytotoxicity. Additionally, the ferromagnetic behavior of iron remains an essential topic for further experimental research. Zinc-based biodegradable alloys offer a compelling alternative to magnesium and iron counterparts, with increased biocompatibility and moderate degradation behavior (electronegative potential of − 0.76 V)^10,11^. Zinc is important element for physiological bone homeostasis and pathological bone turnover^12^. O’Connor et al.^13^ and Zhang et al.^14^ showed that zinc can provide increase in bone cells proliferation, differentiation and mineralization. Su et al.^15^ highlighted zinc's importance in metabolism and immune processes. However, the use of pure zinc as a biodegradable material is limited due to its insufficient strength and hardness. The Zn–Mg alloy combines biocompatibility, corrosion resistance, mechanical strength, controlled degradation, low toxicity, and cost-effectiveness, making it promising for biomedical application. Li et al.^16^ showed that binary Zn–1Mg alloy exhibits sufficient mechanical support during the tissue-repair process. Kubásek^17^, through in vivo testing involving the placement of hemispherical implants into a rat cranium, demonstrated that Zn–2Mg alloy had no detrimental effects on the behavior or physical condition of the rats. Furthermore, there were no observed gas bubbles or inflammatory reactions in the living tissue after the 12 weeks. Surface modification methods hold promise for improving the properties of zinc-based materials^18^. Various types of protective coatings have been used in the literature to achieve the appropriate degradation progress of zinc alloys. Additionally, surface modification can be employed to generate a conducive surface morphology for cell proliferation or alter the surface chemistry to mitigate inflammation after surgical procedures. For short-term implants based on biodegradable metal alloys, the deposition of biodegradable organic and polymer coatings can ensure superior biomaterial properties. Su et al.^19^ applied a Zn–P coating onto the surface of pure zinc. The results indicate that the Zn–P coating significantly enhanced the adhesion, viability, and differentiation of preosteoblasts. Shi et al.^20^ indicated reduced Zn ions release in effect applied PLA coating. Also, Li et al.^21^ demonstrated the effective blocking of zinc ion release from the substrate through the deposition of ingredients similar to the chemical composition of human bone (Ca-P). The study^22^ also contains test results for a coating composed of polycarbonate and heparin on zinc alloys. An electrophoretic deposition (EPD) method improved biocompatibility and provided anticoagulant functions for zinc alloy stents.

Polylactide (PLA), a commonly used biodegradable polymer, offers implants that degrade over 1.5–2 years^23^. However, lactic acid released from PLA coatings can influence the inflammatory response. Lactic acid contributes to a decrease in pH as it accumulates. This change in local pH can influence the inflammatory microenvironment, potentially impacting immune cell function and cytokine (depends on implant size and implantation site)^24^. Shi et al.^25^ showed, that introducing Mg(OH)^2^ submicron particles into poly (L-lactic acid) (PLLA) matric, resulted in reducing the corrosion of the Mg matrix but also increasing cell proliferation.

Surface functionalization with biologically active molecules can improve PLA's surface properties. Casein, with approximately 6% calcium phosphate^26^ was used as the second component of the new coating material. Casein is biocompatible and well tolerated by the body, even in high concentrations, so that it can be used for many biomedical applications. Additionally, casein has specific properties that make it very suitable for drug delivery applications^27^.

Our study aimed to develop innovative PLA-based coatings with the addition of casein to improve the properties of biodegradable ZnMg1.2 wt% alloy. The changes in surface properties of PLA films after modification were investigated to determine the impact of a natural biopolymer, i.e. casein, on the degradation control process, the morphology and degradation course of a zinc alloy sample covered with a polylactide-casein coating were examined. For comparative purposes, a sample of a zinc alloy with a coating consisting only of a synthetic polymer, i.e. polylactide was prepared and investigated.

## Materials and methods

The ZnMg1.2 wt% zinc alloy was the substrate material for the coatings. The test samples were produced by precision casting of master alloy. The master alloy was obtained by induction melting in a ceramic crucible (Al_2_O_3_) of Zn and Mg elements with a purity of 99.95%. Magnesium was melted in liquid zinc and heated to its melting point. Casting temperature was 500 °C. Casting part was in the form of a cylinder with dimensions of 10 mm in diameter and 50 mm in length.

Polymer coatings were obtained by preparing solutions consisting of 0.5 g of polylactide granules (purity 99%) and 20ml of dichloromethane (99.5% purity)—the sample was marked as 0.5PLA. The second coating was prepared by mixing with a magnetic stirrer 10 ml of a solution consisting of 0.5 g of polylactide granules (purity 99%) and 20 ml of dichloromethane, and 2 ml of a solution consisting of 20 ml of distilled water, 2 g of casein, 4 g of NaOH—the sample was marked as 2CAS + 0.5 PLA. Then, the ZnMg1.2 wt% alloy was immersed in the prepared solutions three times for about 10 s using the dip coating method and left to dry at room temperature.

Structural examinations of zinc alloy were performed using a Zeiss optical microscope at 200× magnification. Observations of the surface of the studied materials were made using a SEM Zeiss Supra 25 scanning electron microscope. To observe samples with coatings, it was necessary to cover them with a layer of gold-based conductive material. The geometric structure of the sample’s surfaces was analyzed using a Leica DVM6 digital microscope (DM).

The adhesion of tested coatings to ZnMg1.2 substrate material was examined using a Pull-off Adhesion AT-A20 tester by PosiTest. The dolly (stub) with 20 mm diameter was affixed with cyanoacrylate glue. The force required to pull the dolly off yields the tensile strength (MPa). The surface of the stub and coatings surface after the test were examined using a stereoscopy microscope SteReo Cl 1500 ECO by Zeiss. For both coatings, five measurements were taken.

Immersion tests of the substrate sample and Zn alloy samples with coatings were performed in Ringer's solution (chemical composition: NaCl = 8.6 g/dm^3^; KCl = 0.3 g/dm^3^; CaCl_2_·6H_2_O = 0.48 g/dm^3^ at 37 °C for 168 h. In order to examine the degradation process of the Zn alloy substrate sample, the degradation products were removed in an aqueous solution of chromic acid anhydride. 50 g of chromic anhydride and 200 ml of distilled water were used to prepare the solution. The ingredients were mixed at room temperature.

Electrochemical test (Tafel plot and electrochemical impedance spectroscopy) were performed using an AUTOLAB 402N potentiostat and a three-electrode measurement station. The reference electrode was Ag/AgCl^28^. The measurement time of the open circuit potential was 1 h in Ringer's solution at 37 °C. Using the NOVA software version 1.11, the corrosion potential (E_corr_), corrosion current density (i_corr_), polarization resistance (R_p_) (Eq. 1) and corrosion rate (V_corr_) (Eq. 2) were all calculated. The potentiodynamic polarization is measured by the Stern Geary equation.

1 $${R}_{p}=\frac{1}{2.303}\cdot \frac{{b}_{a}\cdot {b}_{c}}{{b}_{a}+{b}_{c}}\cdot \left(\frac{1}{{i}_{corr}}\right)$$

2 $${R}_{M}=3.17E-9\frac{M}{nF\rho A}{i}_{corr}$$

where R_p_ (Ω)—polarization resistance, b_a_, b_c_ (V)—anodic and cathodic Tafel constants, respectively, i_corr_ (A/cm^2^)—corrosion current density, 3.17E-9—the conversion from cm s^−1^ to mm year^−1^; M (g mol^−1^)—the atomic weight of the samples, n—the number of the electrons exchange in the reaction (M/n—equivalent weight), ρ (g cm^−3^)—density of the samples; F (96458 C mol^−1^)—the Faraday constant, and A (cm^2^)—the area of the samples

For EIS analysis, the applied frequency range was 10^4^ to 10^–3^ Hz, and the amplitude was 5 mV of AC voltage. The impedance spectra in the form of the Nyquist and Bode diagrams were determined, and next obtained data were adjusted to the equivalent circuit using the least-squares method.

Contact angle (Θ) measurements were carried out using the sitting drop method to determine the wettability of the tested samples surface. For this purpose, the Biolin Scientific Attension Theta Flex strain gauge was used. Drops of distilled water as measuring liquid in the volume of 2 µl were deposited on the surface of the tested samples, each time maintaining the minimum (same) height of the dispenser above the surface. Contact angle measurements were performed as a function of time (60 s) in a series of 5 measurements for each sample. Measurements were carried out at room temperature 289 K (25 °C).

To identify the functional groups existing on the coating's surface before and after immersion in Ringer's solution the Fourier-transform infrared spectroscopy was applied. FTIR-ATR spectra were recorded on the Nicolet 6700 FTIR spectroscope with the GladiATR adapter. The samples were investigated in a mid-infrared range of 4000–400 cm^−1^.

## Results

### Studies of substrates

**Figure 1:**
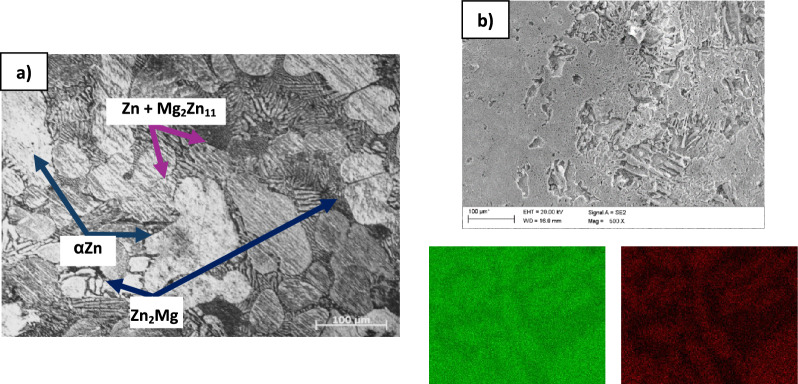
The structure of as cast ZnMg1.2 wt% alloy at ×200 magnification **(a)** and surface of ZnMg1.2 wt% after removal of corrosion products **(b)**.

Structural studies of ZnMg1.2 wt% alloy (Fig. 1a) indicated a multiphase structure. According to the Zn-Mg phase diagram, the microstructure of the as-cast binary alloy is composed of Zn (light) and ZnMg_2_ (dark) partially transformed dendritic grains and lamellar Zn + Mg_2_Zn_11_ eutectic (matrix). Similar observation showed Yang et al.^29^. As indicated by Liu et al.^30^ the columnar grain structure of pure Zn can be transformed into equiaxed grains by the introduction of even minor Mg (Mg has very limited solubility in the Zn matrix).

The surface morphology of ZnMg1.2 wt% alloy after 168 h of immersion tests was given in Fig. 1b. To remove the degradation product chromic acid anhydride was used. Surface morphology tests revealed nonuniform degradation of the alloy, with visible defects observed in the substrate material. When there is uneven progress in the degradation of a ZnMg-based implant, an increase in the risk of premature failure is possible due to the compromise of their mechanical integrity. The uneven degradation may lead to an inconsistent release of degradation by-products in the surrounding tissue. This can result in varying degrees of inflammatory response and tissue reactions, potentially affecting the healing process. It was the premise for carrying out tests on coated samples of the ZnMg1.2 wt% alloy.

### Studies of coated samples

**Figure 2:**
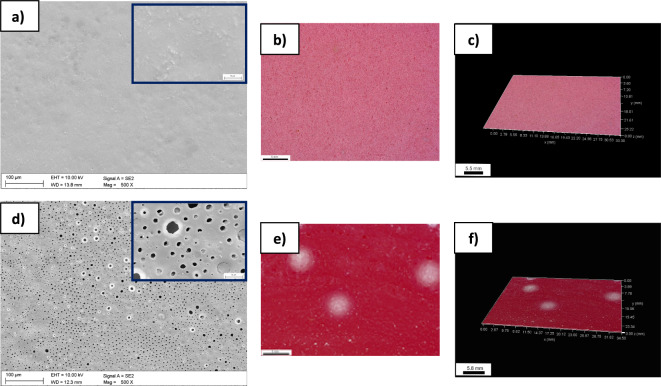
Surface morphology analysis 2CAS + 0.5PLA coating: SEM, SE **(a)**, DM **(b, c)** and the 0.5PLA coating: (SEM, SE) **(d)**, DM **(e,f)** on the ZnMg1.2 wt% alloy.

The results of the morphology observation the produced polymer coatings 2CAS + 0.5PLA (Fig. 2a) and 0.5PLA (Fig. 2d) on the ZnMg1.2 wt% zinc alloy indicate a different structure of both types of coatings. The 2CAS + 0.5PLA coating is homogeneous, although it contains irregular elements (roughness). The rough structure probably results from the massive polar, nonpolar, and the peptide chain self-aggregation during the drying process. The digital microscopic analysis (Fig. 2b,c) of 2CAS + 0.5PLA confirmed the, SEM observations. The coating was shown to be homogeneous and uniform. The presence of surface defects was not visible. In the case of samples made of pure 0.5PLA, microscopic observations (SEM,DM) confirmed the presence of an irregular coating suvrface with numerous bubbles and pores distributed throughout the entire volume of the analyzed layer (Fig. 2d–f). The analysis also showed that the 0.5PLA sample is characterized by increased surface development compared to the 2CAS + 0.5PLA samples. It was found that the addition of casein to the PLA matrix leads to the gradual filling of pores and smoothing of surface morphology, indicating enhanced continuity and compactness of the composite films. According to the works presented by Kang et al.^31^, this improvement could be attributed to imino groups, which enhance the interfacial compatibility between casein and PLA.

**Figure 3:**
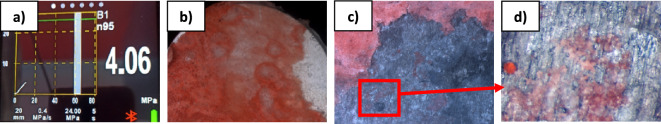
Results of adhesion test for 2CAS + 0.5PLA samples group: view of pull-off tester **(a)**, dolly surface after test **(b)**, ZnMg1.2 substrate material surface after the test **(c)** and **(d)**.

The pull-off test assessed the strength bonding of coatings to ZnMg1.2% Substrate. It was found that the maximum values of the yields the tensile strength were 7.6 and 5 MPa for 0.5PLA and 2CAS + 0.5PLA samples group respectively. Based on microscopic observation (Fig. 3b–d), it was shown that the bond failure was adhesive (failure at the coating/substrate interface).

**Figure 4:**
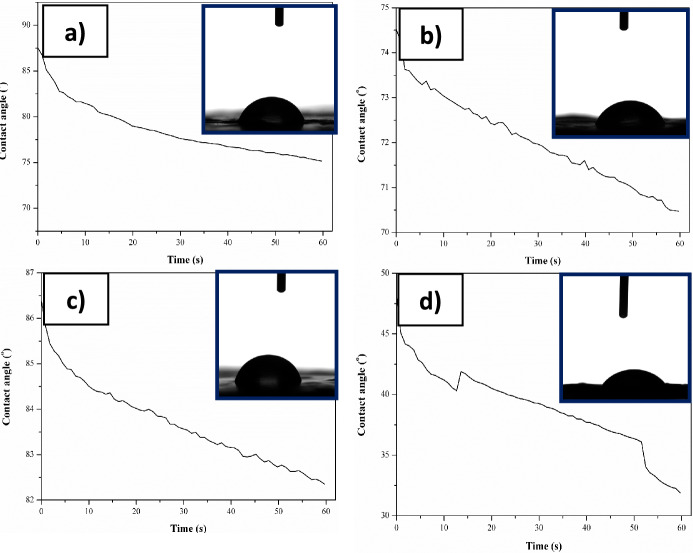
Examples image of distilled water drops on tested materials and diagram of changes of contact angle in 60 s in initial state: 2CAS + 0.5PLA **(a)**, 0.5PLA **(b)**, and after immersion test 2CAS + 0.5PLA **(c)**, 0.5PLA **(d)**.

Based on the results of the wettability test (Table1, Fig. 4), it was observed that the water contact angle of the ZK60 substrate material is approximately 77° (the measured values were less than 90°.), indicating a weakly hydrophilic surface typical for Mg-based alloys in an oxygen atmosphere^32^. In addition, it was found that surface modification doesn't effect on the biomaterial chemical surface character. For samples in the initial state, both 2CAS + 0.5PLA and 0.5PLA, the hydrophilic nature of the surface was demonstrated. Additionally, samples with a 2CAS + 0.5PLA coating exhibit a higher contact angle, with an average value of approximately 77°, compared to those with a 0.5PLA coating. As indicated by Yavari et al.^33^ for the electrospun polycaprolactone/casein nanofibers, added caseinas the hydrophilic segment in the PCL (in our work PLA), due to the presence of carboxyl and amine groups along the main polymer chain, changed the surface wettability. Casein exhibits a low contact angle of 34°. However, in the composite PLA + CAS structure, the morphology may become more homogeneous and compact. It is possible that a three-dimensional rigid network forms, which could close some hydrophilic groups on the casein surface, thus preventing water from permeating the surface of the films^34^. The analysis of changes in the contact angle as a function of 60 s for both groups of samples with the polymer coatings in the initial state indicates the stability of the measurement liquid drops over the entire measurement range. Exposure to Ringer's solution for 168 h did not significantly change the degree of wetting for the 2CAS + 0.5PLA samples. The recorded average contact angle was 77°, which is the same value recorded for the initial condition. In turn, for 0.5PLA samples, a decrease in the contact angle was noted after exposure to Ringer's solution. The measured average value was 41°, which is more than 1.7 times lower than that recorded for the initial samples.

**Table 1:** Results of distilled water contact angle measurements.

Name		Θ (°)
Initial state	ZnMg1.2 wt%	77.2 ± 4.2
	2CAS + 0.5PLA	76.6 ± 3.0
	0.5PLA	69.2 ± 1.5
Immersion 168h	2CAS + 0.5PLA	76.8 ± 6.0
	0.5PLA	41.2 ± 9.0

**Figure 5:**
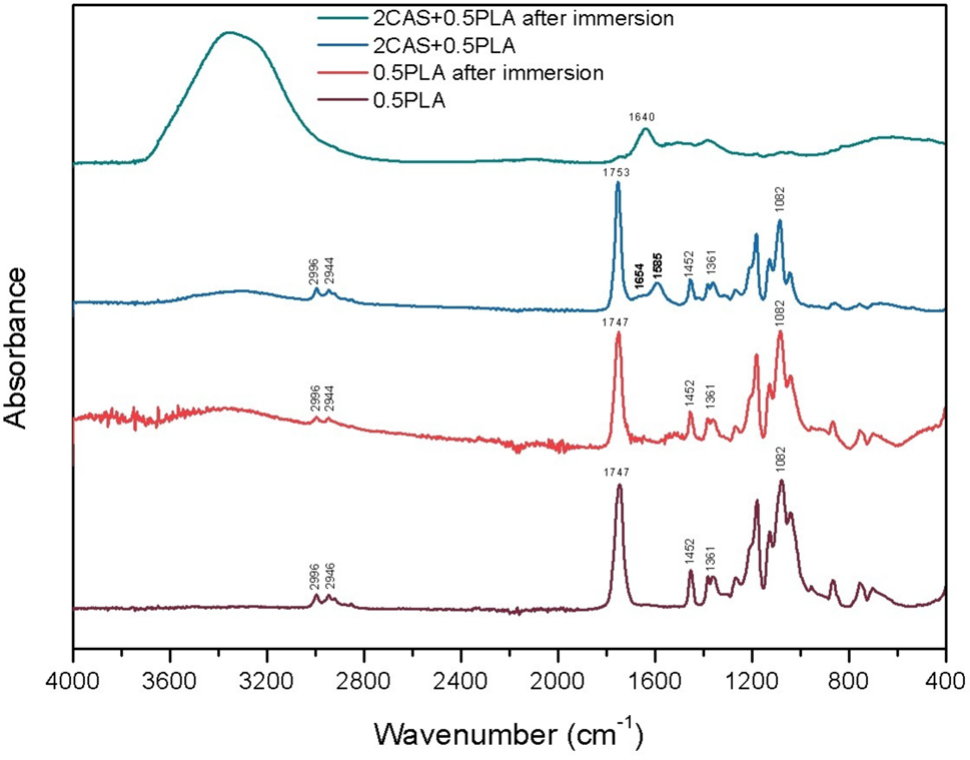
FTIR spectra before and after immersion test in Ringer’solution at 37°C.

In order to identify and compare the bonds and functional groups present on the surface of the 0.5PLA and 2CAS + 0.5PLA coatings before and after 168 h of immersion in Ringer's solution, the FTIR technique was used (Fig. 5). Analysis of the spectrum of the 0.5PLA sample before immersion showed the presence of an absorption band characteristic of PLA at 1747 cm^−1^, which is associated with the stretching vibrations of the C=O group. The bands at 2996, 2946, 1452 and 1361 cm^−1^ are associated with symmetric and asymmetric stretching vibrations of CH_3_ groups, while the band at 1081 cm^−1^ can be assigned to the stretching vibrations of C–O groups^23,35^. In the case of the 0.5PLA coating after immersion, in addition to the absorption bands characteristic of PLA, a broad band at 3000–3600 cm^−1^ was also observed, indicating the adsorption of water molecules from the solution on its surface. The FTIR spectrum of the 2CAS + 0.5PLA sample before immersion has bands characteristic of both PLA and two main bands confirming the presence of casein at 1654 and 1585 cm^−1^, which can be assigned to the amide I (C=O) and amide II (N–H, C–N), respectively^36,37^. Moreover, compared to the spectrum of the 0.5PLA sample without immersion, a broad band in the range of 3600–3000 cm^−1^ is visible, indicating the presence of water. This is due to the way casein was added to PLA—in the form of an aqueous solution with the addition of NaOH. The spectrum recorded for the 2CAS + 0.5PLA coating after 168 h of immersion shows a broad band at 3600–2900 cm^−1^, representing O–H stretching vibrations. Moreover, the presence of O–H–O bending scissors at 1640 cm^−1^ was found, which, together with the second observed band, allows us to conclude that only water was adsorbed on the surface of the 2CAS + 0.5PLA sample after immersion^38,39^.

**Figure 6:**
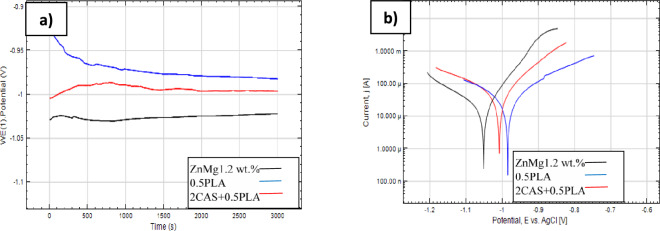
E_*OCP*_ measurement results **(a)** and potentiodynamic curves **(b)** of coated samples and Zn1.2Mg wt% alloy.

In order to determine the influence on the corrosion behavior of the coatings, open circuit potential (E_*OCP*_) measurements were performed, and potentiodynamic curves were recorded after 1 h of immersion in Ringer's solution at 37°C (Fig. 6). For the uncoated Zn alloy sample, the lowest open circuit potential, but stable over the entire measurement range with minor deviations. According to the Proubaix diagram^40^, registered value of the E_OCP_ for Zn-based substrate material (− 0.8 V vs. NEH) belongs to Zn^2+^(aq) or active ZnCl^+^ domain, pointed to active degradation of the material. In fac, the phenomenon of zinc passivation occurs within a specific pH range 9–11. Nevertheless, carbon adsorption onto the zinc surface can extend the passive zinc region's boundaries to a pH range of 6 to 11. In addition, chloride ions (from the corrosion medium) shift the passivity region towards higher pH regions, causing dissolution.

Based on the registered E_*OCP*_ curves for the sample with 0.5PLA coating, there is a visible tendency towards higher potential values, i.e. decreasing corrosion resistance. After about 15 min of immersion, the open circuit potential stabilizes. The opposite tendency was demonstrated by the Zn alloy sample with a 2CAS + 0.5PLA coating. A lower potential was recorded in the initial stage (up to approximately 15 min) and then the potential stabilized in the further part of the measurement range (Fig. 6a). Obtained E_OCP_ values of all tested samples belong to biological standard reduction potentials (~ 820 mV to ~  − 670 mV vs. NEH)^28^.

Registered corrosion potentials (E_corr_) confirm E_OCP_ behavior – E_corr_ of the samples with polymers-based coating was slightly more noble as compared to the Zn-based substrate material. Based on the registered Tafel plots (Fig. 6b and Table 2), it was found that for all tested samples the cathodic branches present relatively similar slopes. Anodic branches show a linear segment noticeable, indicating the breakdown of the protective oxide layer formed on the surface of the base material, and is much more visible for the samples in the initial state (uncoated ZK60). Similar observation for the biodegradable Mg-based alloy was observed by Leleu et al.^41^. The b_a_ and b_c_ coefficient values are reported in Table 2. For all samples, it was found that b_a_ values were above the b_c_ ones, which indicates that the oxidation reaction is more sensitive to changes in the overpotential than the reduction reaction. In addition, Tafel`s plot shows anodic current densities (i_a_) are in the same order of magnitude, independently of the material. However, slightly lower values of the i_a_ were registered for Zn1.2Mg and 0.5PLA compared to the 2CAS + 0.5PLA samples, which could suggest that the protective layers on those samples are more protective. However, the values were closer and the surface morphology of the samples after degradation and corrosion test must be taken into account. The 0.5PLA and 2CAS + 0.5PLA sample groups exhibit higher cathodic current densities (i_c_) than those measured for the pure ZK60 alloy. This indicates that water reduction kinetics are increased in the case of the samples after surface modification. Also, the calculated corrosion current densities (i_corr_) and corrosion rate (V_corr_) of the coated specimens were slightly higher than the bare specimen (Fig. 6b and Table 2).

**Table 2:** Results of Tafel’s analysis.

Name	E_corr_ (V vs. Ag.AgCl)	b_a_ (V vs. Ag.AgCl)	b_c_ (V vs. Ag.AgCl)	i_corr_ (µA/cm^2^)	R_p_ (Ωcm^2^)	V_corr_ (mm/year)
ZnMg1.2	− 1.05	290	160	40	998	0.98
0.5PLA	− 0.98	238	177	47	900	1.09
2CAS + 0.5PLA	− 1.00	288	150	48	882	1.10

**Figure 7:**
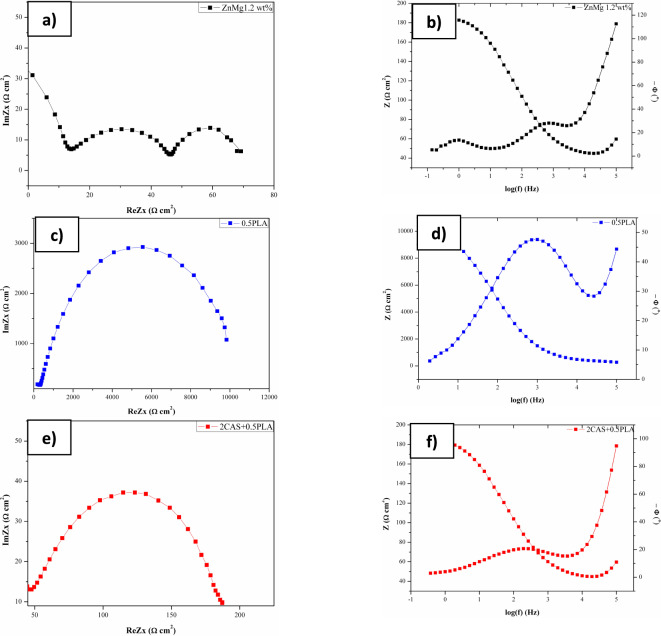
Results of EIS test in the form of Nyquist and Bode diagrams for: ZnMg1.2 wt% **(a)** and **(b)**, 0.5PLA **(c)** and **(d)**, 2CAS + 0.5PLA **(e)** and **(f)**.

Due to large discrepancies in the scale of the recorded curves, the Nyquist and Bode modulus results for materials are presented singly. Registered Nyquist diagram (Fig. 7a,c,e), could suggest that 0.5PLA coating initially acted as a protective film over the metal surface, which indicated also Wang et al. 41. For the uncoated Zn-based alloy, the Nyquist diagram consisted of two semicircles with a tail (three-time constants), and first and second capacitive arcs refers to the oxide passive layer and the charge transfer reactions occurring at the surface. The tail at lower frequencies suggests diffusion-related processes, indicating that the uncoated alloy is subject to both surface and bulk degradation processes. For the 0.5PLA and 2CAS + 0.5PLA samples, single constants were recorded, witch point to a dominant charge transfer resistance without significant diffusion effects, suggesting a more stable and protective coating layer. The Bode phase angle plots further corroborate these findings. The higher phase angles for the coated samples (up to 45° for 0.5PLA) indicate a more capacitive behavior, typical of an intact and protective barrier layer. The broader range of high phase angles for the uncoated alloy suggests variable and less effective protection, correlating with the presence of multiple degradation mechanisms.

**Figure 8:**
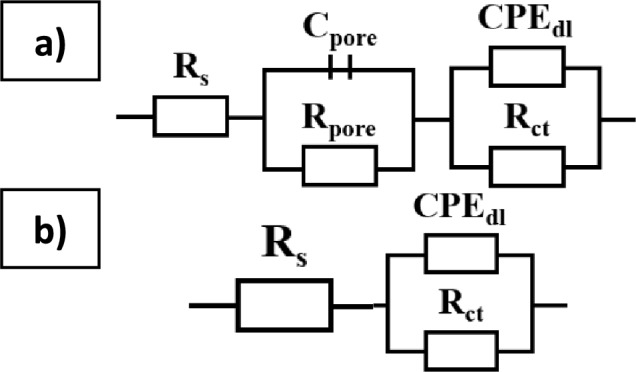
Electric substitute scheme and mathematical impedance models: ZnMg1.2 wt. % **(a)**, 0.5 PLA and 2CAS + 0.5PCL **(b).**

Characterization of the interface impedance of tested samples was done by approximating the EIS experimental data using physical electrical models of the equivalent circuits. For the uncoated samples ZnMg1.2 wt%, an equivalent circuit with two-time constants was used to analyze the EIS data (Fig. 8a), consisting of solution resistance (Rs), the resistance of the porous layer (R_pore_) and capacity of that layer (C), and the resistance of conformal layer (R_ct_), and capacity of that layer (CPE_dl_). For 2CAS + 0.5PLA and 0.5PLA samples an equivalent circuit with a one-time constant was used, represented by a single layer (Fig. 8 b).

The numerical values of EIS tests provide quantitative support for these observations. For the uncoated ZnMg1.2 alloy higher R_pore_ values than the R_ct_ were obtained, which points out that the porous external layer significantly affects the corrosion behavior of samples. The charge transfer resistance (R_ct_) values, which indicate the resistance to corrosion processes at the metal-coating interface, were significantly higher for the coated samples than the uncoated ones. Specifically, the R_ct_ value for the uncoated ZnMg1.2 alloy was measured at 21 Ω·cm^2^. This relatively low R_ct_ value indicates a higher susceptibility to corrosion, as the uncoated surface provides minimal resistance to the charge transfer processes involved in corrosion reactions.

In comparison, the 0.5PLA coating exhibited an R_ct_ value of 1000 Ω·cm^2^, which suggests that the PLA coating forms a more effective barrier, reducing the rate of corrosive species reaching the alloy surface. The improvement in R_ct_ highlights the effectiveness of the PLA coating in mitigating the corrosion processes. The 2CAS + 0.5PLA coating demonstrated the R_ct_ value at 200 Ω·cm^2^. These values indicate that the PLA-casein coating significantly enhances the corrosion resistance of the ZnMg1.2 alloy.

In conclusion, the EIS results demonstrate that the application of PLA and PLA-casein coatings significantly improves the corrosion resistance of the ZnMg1.2 alloy. The higher R_ct_ values and more favorable Nyquist and Bode plots for the coated samples indicate that these coatings provide a more consistent and effective barrier against corrosion. This enhanced performance of samples with polymer coatings, offers the highest resistance to charge transfer, making it the most promising candidate for improving the longevity and biocompatibility of ZnMg1.2 alloy implants (Table 3).

**Table 3:** Results of EIS analysis.

Name	CPE_dl_		R_dl_ (Ω)	C_pore_ (mF)	R_pore_ (Ω)
	Y_1_ (nF)	n			
ZnMg1.2 wt%	–	–	21	6.4	159
0.5PLA	276	0.99	1040	–	–
2CAS + 0.5PLA	21	0.99	200	–	–

3 $${\text{Z}}={\text{R}}_{\text{s}}+\frac{1}{\frac{1}{{\text{R}}_{\text{pore}}}+\text{j}\omega {\text{C}}_{\text{pore}}}+\frac{1}{\frac{1}{{\text{R}}_{\text{ct}}}+{\text{Y}}_{d\text{l}}{\left({\text{j}}\omega \right)}^{{\text{n}}_{\text{dl}}}}$$

4 $$Z={R}_{s}+\frac{1}{\frac{1}{{R}_{Ct}}+{Y}_{01}{\left(j\omega \right)}^{{n}_{2}}}$$

**Figure 9:**
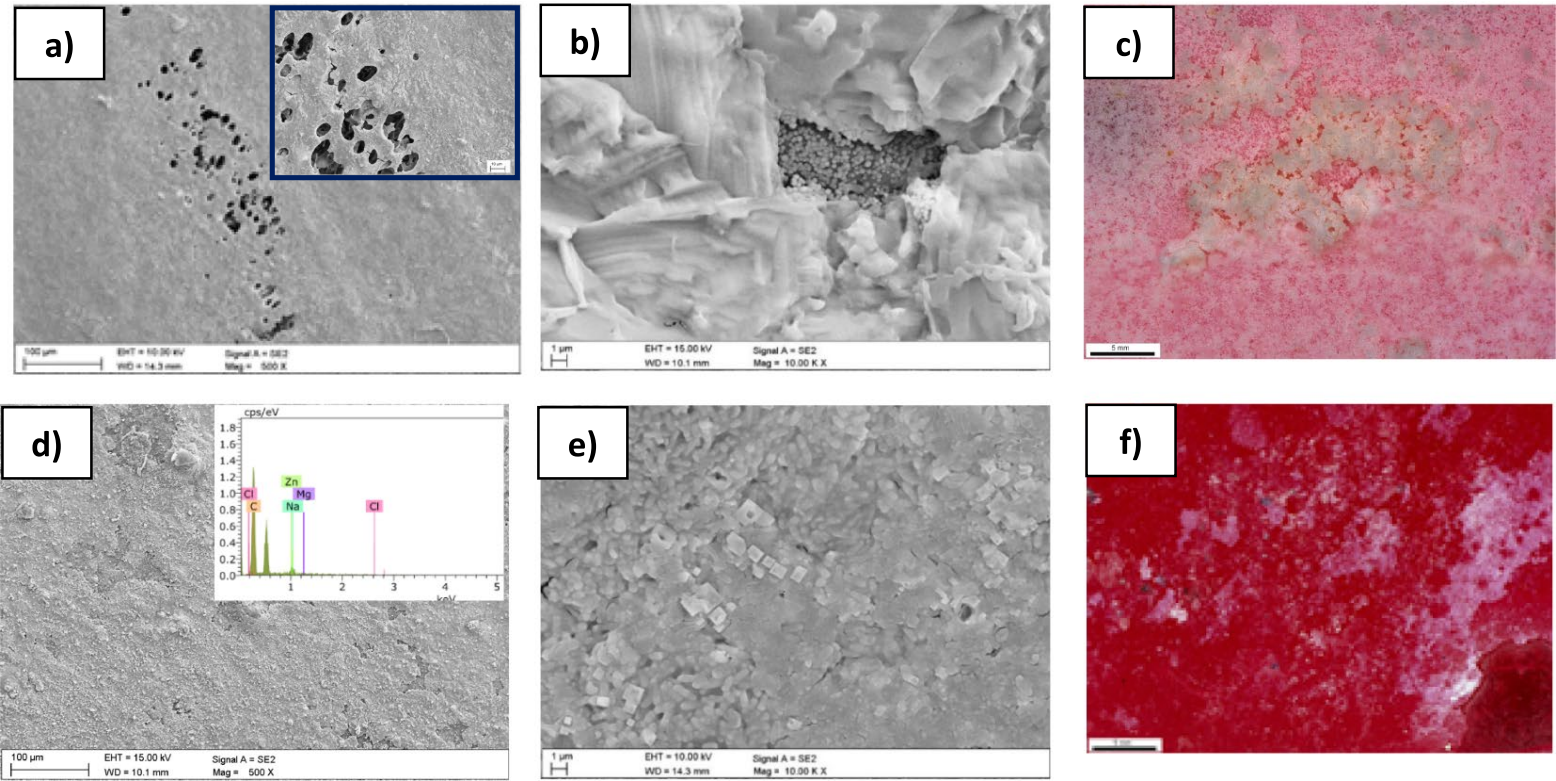
SEM images of the morphology of the 2KAZ + 0.5PLA: SEM,SE **(a,b)**, DM **(c)** and 0.5PLA: SEM, SE **(d,e)**, DM **(f)** coating on the ZnMg1.2 wt% alloy after 168 h of immersion in Ringer's solution at 37 °C.

SEM and DM images show the surface morphology of samples with a 2CAS + 0.5PLA coating (Fig. 9a–c) and 0.5PLA after 168 h of immersion in Ringer's solution at 37 °C. Figure 9a shows circular defects in the coating characterized by a layered structure. Figure 9b shows the areas covered with corrosion products and visible microcracks in the 2CAS + 0.5PLA coating. Figure 9d,e shows the surface of the 0.5PLA coating covered with corrosion products with visible coating losses indicating the coating’s defragmentation. In addition, samples with 0.5 PLA coating exhibit morphologies, consisting of loose, nodular particles, while the 2CAS + 0.5 PLA coated samples had a more compact, plate-like corrosion layer.

## Discussion

**Figure 10:**
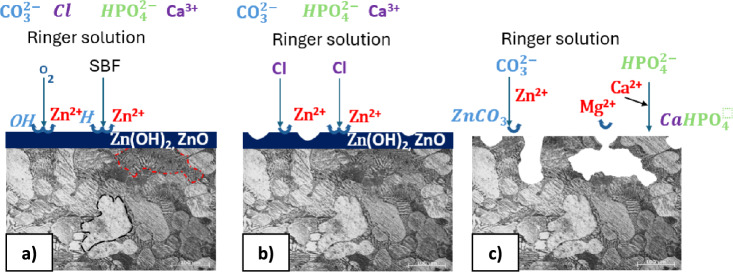
ZnMg alloy degradation scheme: phase first (**a),** second stage (**b)**, and last stage (**c)**. *The grain boundaries are marked by black dashed and the eutectic structures are marked by red dashed for convenience.

In our work, the ZnMg1.2 wt% alloy exhibits a multi-phase structure, which could cause heterogeneous degradation and in effect may adversely affect the integrity and load-bearing capacity of future implants. Generally, the corrosion behavior of as-cast ZnMg alloy is primarily influenced by grain size and eutectic structure. As indicated by Mehrab et al.^42^, the corrosion resistance of biodegradable alloys could be enhanced due to the formation of a low volume fraction of eutectic structures. However, a higher concentration of the secondary phase could generate a greater voltaic potential difference with the αZn phase, thus leading to more corrosion deterioration. The potential difference between the second phase and Zn grain causes galvanic corrosion easily, with the second phase acting as an anode and corroding preferentially^43^. In our work, after 168 h of immersing the alloy sample in Ringer's solution and removing corrosion products, surface defects in the form of pits were visible (Fig. 1). The multi-phase structure is unfavorable in terms of corrosion resistance of metallic materials. In general, the corrosion mechanism of the Zn-based alloys can be presented in three stages^44^. In the initial stage (Fig. 10a), the physiological environment serves as the electrolyte, coming into contact with the Zn matrix and initiating electrochemical reactions. In the anodic reaction, Zn metal dissolves, while the cathodic reaction involves the consumption of oxygen in the corrosion medium (Eq. 5 and 6)^45^:

5 $$ {\text{Zn}}{-}{\text{Zn}}^{{{2} + }} \to {\text{2e}}^{ - } . $$

Cathodic reactions in the case of zinc alloys occur with the consumption of oxygen in the simulating body fluids, according to the following reaction:

6 $$ {\text{2H}}_{{2}} {\text{O}} + {\text{O}}_{{2}} + {\text{4e}}^{ - } \to {\text{4OH}} $$

However, the presence of chloride ions (Cl^−^) in the physiological environment can transform the surface's protective oxide layers into chloride salts. This reduces the protected area, facilitating further material dissolution (Fig. 7b). Additionally, the formation of a galvanic cell between zinc and the second phase (Mg_2_Zn_11_, MgZn_2_) leads to galvanic corrosion. As corrosion progresses, it gradually extends from the initial point, intensifying the corrosion status of the sample (Fig. 10c).

Therefore, it is reasonable to apply protective PLA-based coatings to the surfaces of zinc alloys. Two types of polymer coatings were investigated in this study^46,47^. However, the degradation product of polylactide in the form of lactic acid should be controlled by a regulating agent containing calcium phosphate. Casein meets this condition, and the test results presented in this study indicate that it can change the morphology and degradation course of the polylactide coating. The morphology of the 0.5PLA layer (Fig. 2d–f) is characterized by numerous defects in the form of circular coating defects, probably formed during the evaporation of dichloromethane in which the polylactide granules were dissolved. The analysis of the structure test results also showed that the 0.5PLA sample is characterized by increased surface development compared to samples with casein. In turn, the 2CAS + 0.5PLA coating has no visible defects and is more uniform in terms of structure (Fig. 2a–c). It can be concluded that the method of producing polylactide coatings with the addition of casein developed in this study allowed for the effective combination of both ingredients and obtaining a homogeneous layer.

The results of FTIR analysis of the existing on the surface of the coatings functional groups allowed to confirm the presence of casein and polylactide in the tested materials (Fig. 5). FTIR spectra recorded for coating samples after 168 h of exposure to Ringer's solution indicate absorption on the water surface. It should be mentioned that for the coating with the addition of casein in the position 1730–1720 cm^−1^, no C=O stretching vibrations characteristic of lactic acid were observed, which allows us to conclude that the addition of casein effectively prevents its formation as a result of PLA degradation^48,49^.

The corrosion behavior was determined by performing EOCP measurements Tafel’s plot and EIS analysis to assess the impact of the casein addition on the degradation process (Figs. 6 and 7). It was found that the deposition of 0.5PLA and 2CAS + 0.5PLA coatings leads to increased corrosion resistance.

**Figure 11:**
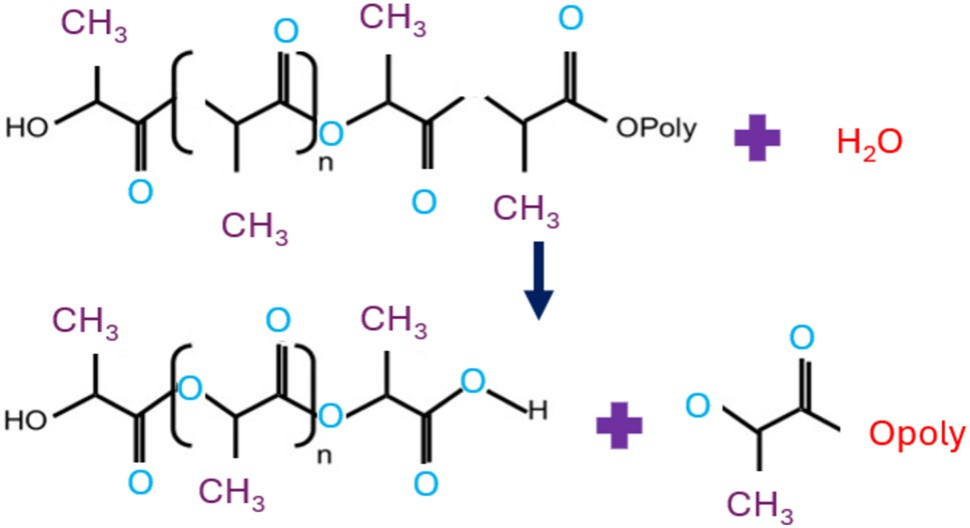
PLA scheme degradation.

The surface morphology changes of the 2CAS + 0.5PLA coating compared to the 0.5PLA coating after long-term exposure to Ringer's solution were also investigated. SEM images (Fig. 9) after 168 h of immersion of samples with the produced coatings show defects resulting from exposure to a solution simulating body fluids. The 0.5PLA coating showed significant degradation progress, or rather defragmentation of the coating, causing significant losses. In general, PLA coating the corrosion enhancement mechanism initiates when the polymer molecules undergo disintegration, and the biodegradation process of PLA (within the human body environment) could be divided into two distinct stages. The initial stage involves hydrolysis, where water permeates the polymer matrix, cleaving ester bonds and resulting in the fragmentation of high molecular weight polyester chains. This hydrolytic process yields small or low molecular weight oligomers along with lactic acid (Fig. 11). These polymer fragments exhibit an acidic nature due to dissociated carboxyl-terminal groups, potentially reducing the pH of their surroundings^50^. Notably, the decrease in environmental pH triggers autocatalysis of the degradation process, as acids accelerate the rate of hydrolysis. The second stage involves the breakdown of PLA by-products, like low molecular weight oligomers, by cellular or microbial metabolism, producing carbon dioxide and water. Acidic molecules formed during PLA degradation, such as lactic acid, corrode metals by reducing the pH around the metal surface, promoting their corrosion, particularly active metals like zinc.

**Figure 12:**
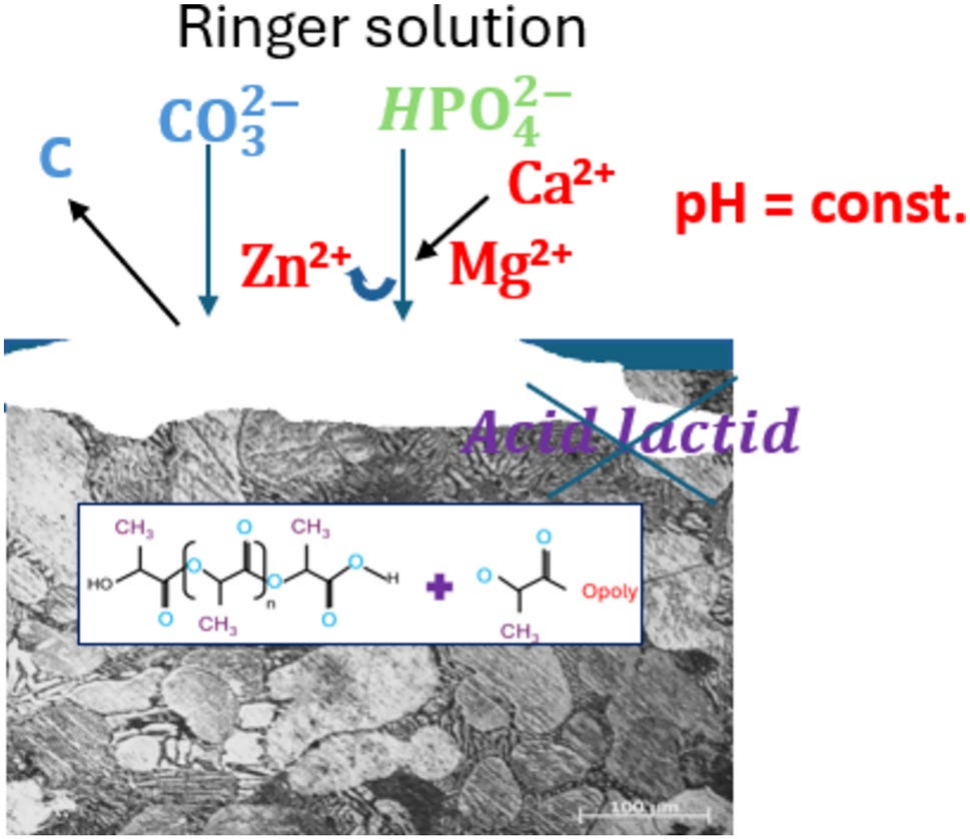
Scheme of possible 2CAS + PLA coating degradation mechanism.

The trend towards higher E_OCP_ values confirms the decreasing protective function of the 0.5PLA coating. While the Nyquist diagram illustrates larger semicircle radii for samples coated with 0.5PLA compared to others, suggesting initial corrosion protection by PLA. The degradation of PLA coatings can be managed by selecting appropriate polymer structure types, and optimal coating thickness. insufficient coating thickness leads to rapid polymer hydrolysis and depletion without significant corrosion acceleration at the metal surface. The opposite was demonstrated by the Zn alloy sample with a casein-added coating, as a lower potential was recorded in the initial stage (up to approximately 15 min), which indicates that the protective properties of the coating increase with increasing immersion time. The 2CAS + 0.5PLA coating also exhibited the existence of surface morphology defects, but they have a layered structure. This is very clearly visible in Fig. 2. Porous structures are desirable because they accelerate the regeneration of bone tissue^51^. Moreover, the location of the anodic part of the potentiodynamic curves in the lower current range for the coated samples indicates a lower activity of anodic reactions than in the case of the uncoated sample (Fig. 12).

In this case, the coated samples show greater activity compared to the uncoated sample. This is directly related to the degradation products of polylactide and casein coatings. After 168 h of immersion, both tested coatings were covered with degradation products in the form of agglomerates, most likely NaCl crystals precipitated from Ringer's solution (Fig. 9). It should also be mentioned that the solubility of casein increases with the progress of hydrolysis^52^. Moreover, the results of FTIR tests also confirm the presence of functional groups that are a product of hydrolysis.

Bakhsheshi-Rad et al.^53^ show that corrosion solution penetrates the coating/Zn alloy interface through pores, initiating corrosion by forming zinc hydroxide. The replacement of OH^−^ by Cl^−^ leads to soluble Zn–Cl compounds, causing localized dissolution and exposing the surface to further corrosion. The ZnMg alloy then degrades as described earlier. The CAS within the PLA coating, with its homogeneous coverage, significantly hinders the transport of corrosive ions (Cl^–^) and electrolytes.

The main goal of adding casein to the PLA matrix is to improve the biocompatibility of the coating by mitigating the inflammatory response typically associated with polylactide (PLA). PLA, while being biodegradable, can produce lactic acid upon degradation, as indicated, by performed FTIR analysis. The release of lactic acid can lower the pH locally, potentially causing an inflammatory response in the surrounding tissues. FTIR analysis confirms that introducing casein to the PLA matrix effectively eliminates the production of lactic acid, thereby reducing the risk of inflammation. In effect, a reduction in the inflammatory response leads to a less aggressive reaction from the human body. Consequently, the degradation process of the coating becomes more uniform degradation process and homogeneous, maintaining a more stable pH level in the surrounding environment. This results in a more controlled and predictable degradation of the implant, enhancing its longevity and performance in the biological environment. The higher and more stable pH levels further contribute to a favorable environment for tissue integration, ensuring that the implant degrades in a manner that supports healing and reduces complications.

The increased adhesive strength likely contributes to the superior corrosion resistance of the single PLA coating. Thicker coatings generally provide better protection, but the integrity and uniformity of the coating are also crucial. The mean thickness of the coatings was approximately 101 and 78 μm, respectively for 0.5PLA and 2CAS + 0.5PLA. The 2CAS + 0.5% PLA samples showed significantly improved corrosion resistance compared to samples in the initial state. Despite slightly less adhesive strength, the addition of casein ensures a more homogeneous degradation profile and eliminates inflammatory responses post-implantation, enhancing biocompatibility. This indicates that while coating thickness is a critical factor, other properties such as layer uniformity, integrity, and the intrinsic properties of the coating materials themselves also play crucial roles in determining the overall corrosion behavior of the ZnMg1.2 wt% alloy substrate. Thus, while the single PLA coating offers the best corrosion resistance, the 2CAS + 0.5% PLA samples provide significant corrosion protection and biocompatibility, making them valuable for specific applications.

In summary, the incorporation of casein into the PLA matrix serves dual purposes: it enhances corrosion resistance by improving the structural integrity of the coating, and it significantly reduces the inflammatory response by eliminating lactic acid production. This makes the polylactide-casein coated ZnMg1.2 alloy a highly promising material for biomedical applications where both durability and biocompatibility are paramount.

The physicochemical properties of the material were also examined, which may also be critical for the interaction of the cell with the material. The surface wettability measured for both coating types indicates the tested materials' hydrophilic nature, which could be an important factor, in quartet good of cell attachment and proliferation on its surface^54^. Increasing the biocompatibility of surfaces in many research environments^55^ involves surface modification of materials to make them hydrophilic. After 168 h of exposure to Ringer's solution, both coatings still showed contact angles indicating the hydrophilic nature of the material. In the case of a coating consisting only of polylactide, this angle decreased from 77° to 41°, which may indicate surface changes due to degradation. However, attention should be paid to the increased value of the standard deviation (2 times higher than for the initial state), which may indicate the local influence of the deposited corrosion products on changes in the contact angle. Hydrophilic polymers create favorable conditions for cells by reducing non-specific or hydrophobic protein adsorption and thus improving cell adhesion^56^.

## Conclusions

The newly developed method of producing polylactide coatings with the addition of casein allowed for the effective combination of both polymers and obtained a homogeneous layer. Compared to a layer consisting only of polylactide, it can be observed that it allows for filling the defects created by the evaporation of dichloromethane. Consequently, the more homogenous casein-polylactide coating is characterized by a more uniform degradation course than the polylactide coating. Unfortunately, after 168 h of immersion in Ringer's solution, the main degradation mechanism is probably hydrolysis of the coating, which increases the solubility of casein, creating defects in the coating. However, due to the hydrophilic nature of the coating surfaces and the layered structure of the defects, it will enable the adhesion and development of cells, which will slow down the degradation processes. Moreover, the newly developed coatings with the addition of casein did not show the presence of groups related to lactic acid, which may solve the problem of acidosis that often occurs when using implants composed only of polylactide.

## Data Availability

All data generated or analysed during this study are included in this published article.
